# Inactive metallopeptidase homologs: the secret lives of pseudopeptidases

**DOI:** 10.3389/fmolb.2024.1436917

**Published:** 2024-07-10

**Authors:** Peter J. Lyons

**Affiliations:** Department of Biology, Andrews University, Berrien Springs, MI, United States

**Keywords:** metallopeptidase, protease, pseudoenzyme, carboxypeptidase, ADAM, AEBP1, mitochondrial processing peptidase, sonic hedgehog

## Abstract

Inactive enzyme homologs, or pseudoenzymes, are proteins, found within most enzyme families, that are incapable of performing catalysis. Rather than catalysis, they are involved in protein-protein interactions, sometimes regulating the activity of their active enzyme cousins, or scaffolding protein complexes. Pseudoenzymes found within metallopeptidase families likewise perform these functions. Pseudoenzymes within the M14 carboxypeptidase family interact with collagens within the extracellular space, while pseudopeptidase members of the M12 “a disintegrin and metalloprotease” (ADAM) family either discard their pseudopeptidase domains as unnecessary for their roles in sperm maturation or utilize surface loops to enable assembly of key complexes at neuronal synapses. Other metallopeptidase families contain pseudopeptidases involved in protein synthesis at the ribosome and protein import into organelles, sometimes using their pseudo-active sites for these interactions. Although the functions of these pseudopeptidases have been challenging to study, ongoing work is teasing out the secret lives of these proteins.

## 1 Introduction

Enzyme families expand through the duplication and subsequent mutation of progenitor genes. While this process often results in the degeneration of duplicate genes to form pseudogenes, lacking functional open reading frames, duplicate genes may continue to produce functional proteins (Conant and Wolfe, 2008; Copley, 2020). These proteins may retain their original functions, restricted to subsets of tissues in which the progenitors were expressed (subfunctionalization), or at different developmental times or places. In yet other cases, proteins acquire new functions through changes in enzyme substrate specificities or activation mechanisms (neofunctionalization), or through loss of enzymatic activity but maintenance of interactions previously involved in enzyme function or intracellular signaling. Occasionally, these inactive enzymes (pseudoenzymes) acquire novel interactions and therefore novel functions.

Recently it has been appreciated that as many as 10% of proteins within any enzyme family may consist of pseudoenzymes (Ribeiro et al., 2019). Pseudoenzymes are more difficult to study than their enzyme relatives, lacking clear activities to track. Therefore, we are just beginning to learn of their biological and biochemical functions (Zaru et al., 2020). Work to date has shown these functions to include regulation of active enzymes, integration of molecular signals, interaction with protein complexes, and competition for enzyme substrates or enzyme complex assembly (Reynolds and Fischer, 2015; Murphy et al., 2017; Goldberg and Sreelatha, 2023). In some cases, it has been found that certain pseudoenzymes are not pseudoenzymes at all, as new enzymatic functions have been discovered (Sreelatha et al., 2018; Black et al., 2019; Park et al., 2022). Some pseudoenzymes have been implicated in diseases, highlighting the need to understand more about their mechanisms of action (Fukata et al., 2006; Adrain et al., 2012; Reiterer et al., 2014; Cui et al., 2015; Dulloo et al., 2019).

Recent reviews have highlighted the functions of pseudoenzymes from the kinase and phosphatase families (Reiterer et al., 2020; Mace and Murphy, 2021; Mattei et al., 2021; O'Boyle et al., 2022; Goldberg and Sreelatha, 2023). Pseudokinases have co-opted kinase activation mechanisms to initiate allosteric interactions through molecular switch conformational changes and the formation of signaling scaffolds (Mace and Murphy, 2021). Pseudophosphatases function in similar ways (Reiterer et al., 2014; Mattei et al., 2021; Goldberg and Sreelatha, 2023). Less has been said about pseudoenzymes from peptidase families, although some studies have investigated the functions of serine and cysteine pseudopeptidases (Reynolds and Fischer, 2015). Serine pseudopeptidases have been shown to play important roles in fertility, development, and immune responses, and these have been reviewed elsewhere (Reynolds and Fischer, 2015; Zupanic et al., 2023). The iRhoms are inactive members of the rhomboid protease family. While the rhomboid proteases cleave transmembrane substrates for intercellular signaling, the iRhoms bind to transmembrane domains without cleaving them, modifying the trafficking of these membrane proteins from the endoplasmic reticulum (Adrain and Freeman, 2012). Pseudopeptidases within caspase and metacaspase families have also been identified. Metacaspases 1 and 4 within *Trypanosoma brucei* are inactive enzymes that interact with and regulate the functions of their active cousins in parasitic virulence. C-FLIP_L_ is a mammalian caspase 8 homolog that regulates the extrinsic apoptosis pathway through interaction with caspases 8 and 10 (Reynolds and Fischer, 2015).

While the above illustrates the importance of serine and cysteine pseudopeptidases, the largest family of human peptidases is the metallopeptidase family (Puente et al., 2003). Here I review current knowledge regarding pseudoenzymes within the metallopeptidase family, a number of which have been shown to have significant roles in health and disease (Table 1). I consider their biochemical and biological functions, and the specific modifications that have led to their becoming pseudoenzymes (Table 2). Since the majority of pseudometallopeptidases are found within the M12 and M14 families (MEROPS classification), I highlight these.

**TABLE 1:** Pseudopeptidase members of the metallopeptidase family and their known biological functions.

Peptidase family	Pseudopeptidase	Disease connection or biological function	References
M1	Tma108	Protein translation	Delaveau et al. (2016)
M12	unc-71	*C. elegans* axon guidance and sex myoblast migration	Huang et al. (2003)
M12	ADAM2, 7	Sperm maturation	Cho et al. (1998), Nishimura et al. (2001), Choi et al. (2015), Voronina et al. (2019)
M12	ADAM11	Spatial learning, motor coordination, pain transmission and perception	Takahashi et al. (2006a), Takahashi et al. (2006b)
M12	ADAM22, 23	Severe epilepsy, cerebral atrophy	Sagane et al. (2005), Owuor et al. (2009), Muona et al. (2016), Yamagata et al. (2018), van der Knoop et al. (2022)
M13	Gone early	*Drosophila* germ cell development	Matsuoka et al. (2014)
M14	AEBP1	Ehlers-Danlos syndrome	Alazami et al. (2016), Blackburn et al. (2018), Hebebrand et al. (2019), Ritelli et al. (2019), Syx et al. (2019), Di Giosaffatte et al. (2022), Angwin et al. (2023), Sanai et al. (2023), Yamaguchi et al. (2023)
M14	CPXM1	Adipogenesis	Chang et al. (2004), Kim et al. (2016)
M14	CPXM2	Cardiac hypertrophy	Grabowski et al. (2022)
M14	CPD-III	*Drosophila* viability, virus receptor	Eng et al. (1998), Sidyelyeva et al. (2010)
M15	Shh	Pattern formation in development	Himmelstein et al. (2017)
M16	MPP	Mitochondrial signal peptide processing	Pollock et al. (1988), Taylor et al. (2001)
M24	EBP1	Cancer	Stevenson et al. (2020)
M41	FtsHi1-5	Chloroplast protein import	Kikuchi et al. (2018), Mishra and Funk (2021)

**TABLE 2:** Pseudopeptidase substitutions in active site residues characteristic of metallopeptidases.

Inactive enzymes	Zinc-binding^a^	Substrate binding	General base^a^	References^b^
M1 LTA4H	**H**Exx**H**x_18_ **E**	Y378	H**E**xxH	Handbook Ch. 96
Tma108	**H**Exx**H**x_18_ **E**	—	H**E**xxH	
**M12 ADAMs**	**H**ExG**H**xxGxx**H**D	M(Met-turn)	H**E**xGH	Handbook Ch. 248
unc-71	**Q**SIG**H**LLGLE**H**D	M	Q**S**IGH	Huang et al. (2003)
ADAM2	**Q**LLS**L**SMGIT**Y**D	M	Q**L**LSL	
ADAM7	**H**QLG**H**NLGMQ**H**D	M	H**Q**LGH	
ADAM11	**Q**TLG**Q**NLGMM**W**N	M	Q**T**LGQ	
ADAM18	**Q**LLG**L**NVGLT**Y**D	M	Q**L**LGL	
ADAM22	**Q**SLA**H**NIGII**S**D	M	Q**S**LAH	
ADAM23	**Q**SLA**Q**NLGIQ**W**E	M	Q**S**LAQ	
ADAM29	**H**HLG**H**NLGMN**H**D	M	H**H**LGH	
ADAM32	**Q**MLA**L**SLGIS**Y**D	M	Q**M**LAL	
**M13 Neprilysin**	**H**Exx**H**_58_ **E**		H**E**xxH	Handbook Ch. 127
Gone early	**E**Axx**D**_73_ **V**		E**A**xxD	Matsuoka et al. (2014)
**M14 MCPs^c^**	H69,E72,H196	R127,R145	**E270**	Handbook Ch. 289; (McDonald et al., 2020)
AEBP1	H69,E72,N196	L127,E145	Y270	
CPXM1	H69,E72,H196	R127,H145	E270	
CPXM2	H69,E72,Q196	R127,N145	Y270	
CPD-III	H69,A72,D196	Q127,T145	Y270	
Ecm14	H69,E72,H196	R127,H145	K270	
**M15 (D-Ala-D-Ala CP)**	H154,D161,H197	R138	D194	Handbook Ch. 311
Shh	H140,D147,H182	R123	A179	Maun et al. (2010)
**M16 (βMPP)**	**H**xxE**H**x_76_ **E**	—	Hxx**E**H	Handbook Ch. 322
αMPP	**H**xxD**R**x_76_ **D**	Glycine-rich loop	Hxx**D**R	Taylor et al. (2001)
**M24 MAP2**	D251,D262, H331,E364,E459	F219,I338	E364	Ye et al. (2006), Kowalinski et al. (2007)
EBP1	D109,N120, H188,D227,K320	F76,I195	D227	Kowalinski et al. (2007)
**M41 (E. coli FtsH)**	**H**Exx**H**x_74_ **D**	GPPGTGKT199 (ATP-binding)	H**E**xxH	Handbook Ch. 144
FtsHi1	**T**EVG**V**x_80_ **Y**	GPPGCGKT477	T**E**VGV	Mishra and Funk (2021)
FtsHi2	**N**EAA**M**x_78_ **T**	GPPGVGKT457	N**E**AAM	
FtsHi3	**M**P---x_23_-	GPPGTGKT381	M**P**---	
FtsHi4	**R**EAA**V**x_74_ **S**	GPPGTGKT361	R**E**AAV	
FtsHi5	**W**AAG**R**x_81_ **E**	GERGTGKT831	W**A**AGR	

^a^ Key amino acids are bolded if shown within neighboring amino acids.

^b^ Handbook of Proteolytic Enzymes, Third Edition (Barrett et al., 2012) (cited above as “*Handbook*”), is an excellent resource for information on active enzymes. Publicly available databases (i.e., Uniprot, NCBI) were also referenced for this data.

^c^ Amino acid numbers for M14 peptidases are derived from mature bovine CPA1, by convention.

## 2 M12 ADAMs

**FIGURE 1:**
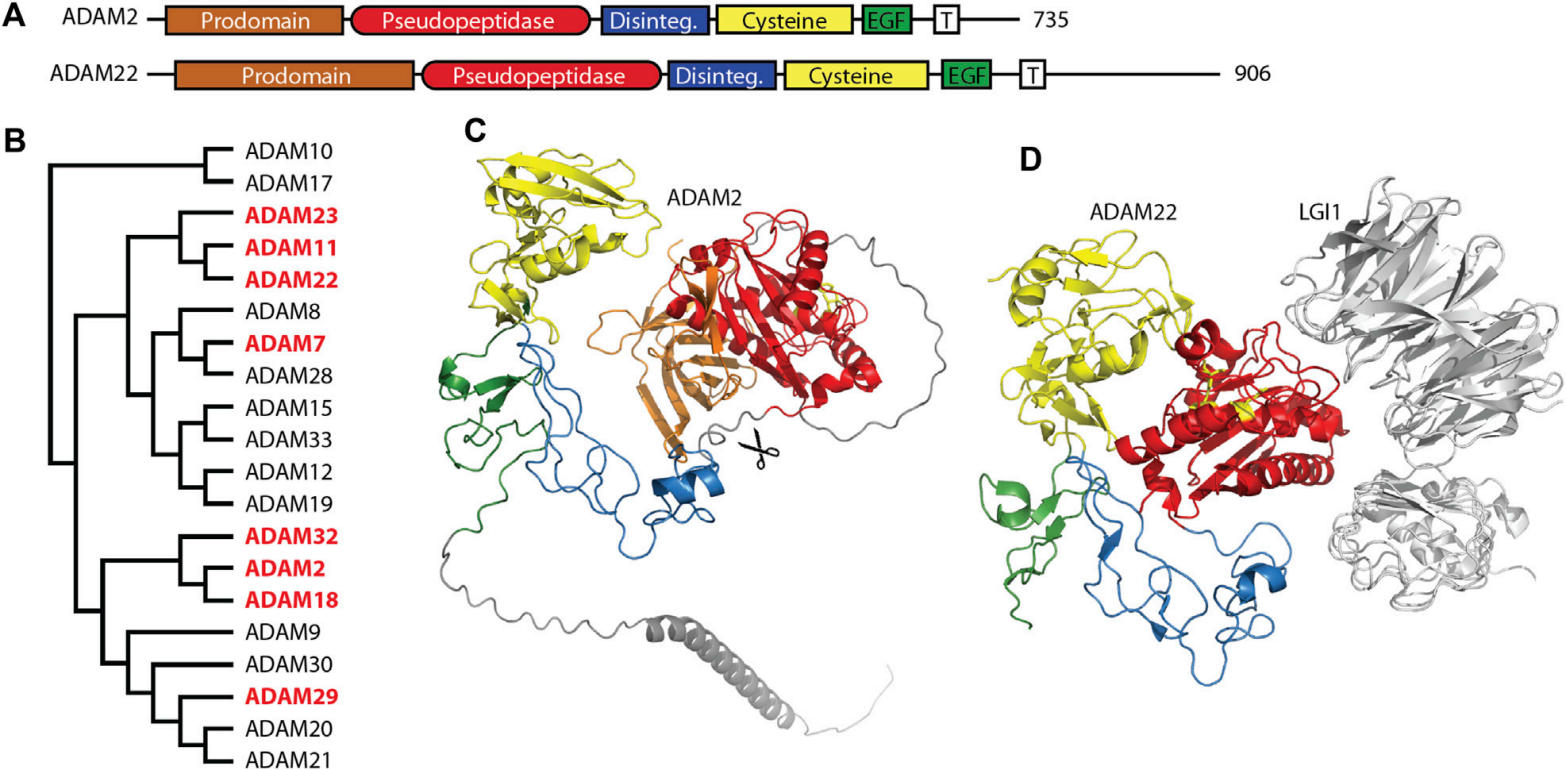
Many members of the M12 ADAM family of metallopeptidases are pseudopeptidases. **(A)** ADAM family members have a very similar domain structure, with an N-terminal prodomain and pseudopeptidase domain followed by a disintegrin domain, cysteine-rich domain, EGF-like domain, and a transmembrane segment leading to a cytosolic C-terminus. **(B)** Protein sequences for all human ADAMs were aligned with Clustal Omega and a midpoint-rooted cladogram prepared with Iqtree2 and Dendroscope. Pseudopeptidase members are shown in red. **(C)** The predicted structure of human ADAM2, as modeled by Alphafold (AF-Q99965-F1-model_v4). Domains are color-coded as shown in **(A)**, scissors indicate the location of proteolytic cleavage, and pseudoactive site residues are shown as yellow sticks. **(D)** The structure of human ADAM22 in complex with LGI1 (5y31), showing that the surface of ADAM22, not the pseudoactive site (yellow sticks), is involved in this interaction. Domains are color-coded as shown in **(A)**.

The ADAM (a disintegrin and metalloprotease) family is a large family of single-pass transmembrane proteins, found within the larger metzincin family of metalloproteases. Metzincins include the matrix metalloproteases (MMPs), ADAMs, ADAM proteases with thrombospondin motifs (ADAMTSs), astacins, and others, all of which contain a zinc within their catalytic sites and a conserved methionine within an active site loop, the Met-turn (Gomis-Rüth, 2009). ADAMs typically have extracellular metalloprotease and disintegrin domains, followed by cysteine-rich and EGF repeat regions, a transmembrane segment, and a C-terminal cytosolic tail (Figure 1A) (Seals and Courtneidge, 2003; Alfandari et al., 2009). The disintegrin domain is typically involved in protein-protein interactions, often as a ligand for integrin receptors during cell adhesion (Bridges et al., 2005). The metalloprotease domain functions in the shedding of a variety of cell surface proteins such as the ephrins and cadherins (Seals and Courtneidge, 2003). A number of pseudopeptidases exist in the ADAM family (Figure 1B) with primary roles in neuronal migration, sperm maturation, and CNS function. These pseudopeptidases have many substitutions of both zinc-binding and general base active site residues (Table 2), although they typically conserve the “Met-turn” characteristic of the enzyme family that appears to play an important structural role (Tallant et al., 2010).

### 2.1 Unc-71/ADAM14, a *C. elegans* pseudoenzyme involved in axon and cell guidance

The *C. elegans* genome contains four ADAM genes (*adm-1*, *adm-2*, *adm-4*, and *sup-17*) and one secreted ADAM-like gene (*mig-17*). The *adm-1* gene, also known as *unc-71* or ADAM-14, was identified in the original *C. elegans* screen as an allele that resulted in uncoordinated movement (Brenner, 1974; Podbilewicz, 1996). It was thought to be an inactive enzyme due to the identity of key active site amino acids (Table 2). A number of loss-of-function mutations were identified in the disintegrin domain that resulted in motor axon guidance and sex myoblast migration defects; a version of UNC-71 that lacked the metalloprotease domain was unable to rescue *unc-71*, suggesting a necessary, although currently unknown, role for this pseudopeptidase in the guidance of cells and growth cones within *C. elegans* (Huang et al., 2003).

### 2.2 ADAM1, ADAM2, ADAM3, ADAM6, ADAM7, ADAM18, ADAM29 and ADAM32, pseudopeptidase-containing proteins necessary for sperm maturation

Many ADAM genes, including many that encode pseudopeptidases, are solely or primarily expressed in the testes and play a role either in sperm maturation and/or fertilization (Cho, 2012). Early work showed the pseudopeptidase-containing proteins ADAM1 and ADAM2 (also known as fertilin α and fertilin β) to form a heterodimer necessary for sperm-egg fusion in mice (Primakoff et al., 1987). Targeted deletion of ADAM2, ADAM3, and ADAM6 resulted in infertile mice (Cho et al., 1998; Nishimura et al., 2001; Voronina et al., 2019). Humans lack functional ADAM1, ADAM3, and ADAM6 genes, and so rely on the remaining ADAM2 gene, which is primarily expressed in the testes (proteinatlas.org).

Molecular studies have shed light on the role of these mouse and human ADAMs. Recombinant forms of ADAM1 that correspond to either the disintegrin-like domain or the cysteine-rich and EGF-like repeat can interfere with sperm-egg binding (Wong et al., 2001). A short 8-amino acid segment of the disintegrin domain of ADAM1 can inhibit the binding of both ADAM1 and ADAM2 to eggs, suggesting the critical component of these proteins for sperm adhesion is found within the disintegrin domain (Wong et al., 2001). Many experiments have shown interactions between ADAMs and integrin heterodimers, including interactions with ADAMs 1, 2, and 3, and have been reviewed elsewhere (Lu et al., 2007). It appears that the disintegrin domain is necessary and sufficient for sperm-egg interactions, as the metalloprotease domains of ADAM1, ADAM2, and ADAM3 are proteolytically removed during sperm development (Figure 1C) (Lum and Blobel, 1997; Eto et al., 2002; Marcello and Evans, 2010). This suggests that the metalloprotease domain is dispensable for the functions of these ADAMs.

While ADAM2 is known to have an important role in human fertility, as do ADAMs 1, 3, and 6 in mice, four additional human ADAM pseudopeptidase genes, ADAMs 7, 18, 29 and 32, are also expressed primarily in the human testes. There have been no experiments reported on the function of ADAM18. Experiments in the mouse model suggest that ADAM32 may not be necessary for fertility, as Adam32-mutant mice had normal fertility, testicular integrity, and sperm characteristics (Lee et al., 2020). Abnormally high expression of ADAM32 was recently observed in hepatoblastoma (Fukazawa et al., 2022); experiments in HepG2 and primary hepatoblastoma cells showed that colony formation, cell migration and invasion, and cell viability were increased upon overexpression of ADAM32 and decreased upon knockdown. Together with other experiments it was suggested that ADAM32 may play a role in apoptosis signaling in these cells. ADAM29, although normally expressed in the testes, has been implicated in a variety of cancers, including colon cancer (Alrubie et al., 2024), esophageal cancer (Song et al., 2014; Wang et al., 2020), breast cancer (Purrington et al., 2014; Zhao et al., 2016), melanoma (Wei et al., 2011), and chronic lymphocytic leukemia (Oppezzo et al., 2005; Kienle et al., 2010).

ADAM7 is closely related to ADAM28 (see Figure 1B), an enzymatically active ADAM highly expressed in the epididymis and lymphocytes (Howard et al., 2001). Cell adhesion assays showed that ADAM7 was recognized by the leukocyte integrins α4β1, α4β7, and α9β1; however, this interaction required only the disintegrin domain (Bridges et al., 2005). Early studies showed that ADAM7 was expressed primarily in the mouse and human epididymis and in the anterior pituitary (Cornwall and Hsia, 1997; Lin et al., 2001). In the mouse epididymis, ADAM7 protein was found to be transferred without proteolytic processing to the surface of sperm, where it functioned as an integral plasma membrane protein (Oh et al., 2005; Oh et al., 2009). Proteomics analysis showed that Adam7 formed sperm membrane complexes with the protein chaperones calnexin and heat shock protein 5, and with the integral membrane protein Itm2b within detergent-resistant membranes (Han et al., 2011). It was proposed that these interactions may play a role in sperm capacitation. Indeed, targeted disruption of the Adam7 gene in the mouse showed decreased male fertility due to histological changes in the epididymal epithelium and abnormal sperm function and morphology (Choi et al., 2015). It is not clear, however, what domains of the ADAM7 protein are necessary for its epididymal and sperm function. While the normal function of ADAM7 is in the epididymis, a study of somatic ADAM mutations present in human cutaneous metastatic melanoma found that ADAM7 was frequently mutated and many of these mutations led to decreased cell adhesion to collagen IV and laminin and increased cell migration (Wei et al., 2011). A number of these mutations were present within the metallopeptidase-like domain, with one occurring at the Met-Turn, suggesting a role for the pseudopeptidase domain in this system.

### 2.3 ADAM11, ADAM22 and ADAM23 in CNS function

ADAM11/MDC, ADAM22/MDC2, and ADAM23/MDC3 contain inactive metallopeptidase domains (Figures 1A, D) and are highly expressed in the brain (Sagane et al., 1998). These three proteins have common roles at the neuronal synapse where they interact with LGI proteins, PSD-95 and voltage-gated potassium (Kv) channels. It is likely that minor differences between these three ADAM proteins lie in differences in interaction affinity and expression patterns. A number of recent reviews have considered their functions in detail (Hsia et al., 2019; Yamagata and Fukai, 2020; Fukata et al., 2021b).

ADAM11 was first identified as a potential tumor suppressor gene, that was subsequently found to be expressed primarily in neurons in the mouse CNS and PNS (Katagiri et al., 1995; Rybnikova et al., 2002). While ADAM11-deficient mice were largely normal, they exhibited some deficits in spatial learning, motor coordination, and pain transmission and perception (Takahashi et al., 2006a; Takahashi et al., 2006b). ADAM22 was found expressed only in the nervous system, and a knockout mouse model showed peripheral neuron hypomyelination with severe ataxia and convulsions (Sagane et al., 2005). ADAM23 was also highly expressed in the CNS and mouse knockout resulted in lethal epilepsy (Owuor et al., 2009).

The primary roles for these ADAM proteins appear to be as membrane-bound receptors for the secreted neuronal *leucine-rich glioma inactivated* (LGI) proteins (Figure 1D). ADAM11, ADAM22, and ADAM23 all interact with both LGI1 and LGI4 at the neuronal synapse (Sagane et al., 2008; Owuor et al., 2009). The ADAM22-LGI1 receptor-ligand pair, scaffolded at the synapse with stargazin by PSD-95, enhanced AMPA receptor-mediated synaptic transmission in hippocampal slices (Fukata et al., 2006). Biallelic mutations in ADAM22 prevented protein expression, LGI1 binding, and/or interaction with PSD-95, and, most importantly, resulted in cerebral atrophy leading to infantile-onset epilepsy with profound delay in intellectual and motor development (Muona et al., 2016; van der Knoop et al., 2022). While one study showed that a mutation of ADAM22 within its disintegrin domain prevented binding of LGI1 (Fukata et al., 2006), recent structural characterization of the ADAM22-LGI1 interaction showed that the interaction involved specific residues on the surface of the ADAM22 metalloprotease domain (Figure 1D) (Yamagata et al., 2018). Mutation of these residues in both ADAM22 and ADAM23 disturbed the interaction with LGI1, while mutation of a nearby cysteine was identified in a patient with progressive encephalopathy, cortical atrophy, and severe epilepsy (Muona et al., 2016; Yamagata et al., 2018).

The details of the interactions mediated by ADAM11, 22, and 23 at the synapse have been explored through mouse models. Kole et al. (2015) showed through immunofluorescence and immunoprecipitation that ADAM11 was highly expressed in cerebellar basket cell terminals where it associated with ADAM22, PSD-95, and Shaker type Kv1 potassium channels. An *Adam11* ^Δ12-18^ mutant mouse lacked expression of these proteins at the basket cell termini that impinged on Purkinje cell initial segments and also lacked the rapid ephaptic inhibition of Purkinje cells normally performed by these basket cell interactions, suggesting that ADAM11 played a key role in organizing synaptic proteins (Kole et al., 2015). Similar results were shown for ADAM22 and ADAM23. ADAM22-interacting proteins were identified in the mouse brain using immunoprecipitation and mass spectrometry, and included LGI family members, ADAM22 family members, presynaptic and postsynaptic MAGUKs such as PSD-95, presynaptic Kv1 channels, and 14-3-3 proteins (Ogawa et al., 2010; Fukata et al., 2021a). Loss of the C-terminal PDZ-interacting motif from ADAM22 via a knock-in approach resulted in lethal epilepsy, and super-resolution microscopy indicated a loss of alignment between pre- and post-synaptic proteins. A careful study of ADAM22 and LGI1 hypomorphic mice showed that 50% of LGI1 and 10% of ADAM22 was sufficient to prevent lethal epilepsy. Importantly, this study demonstrated that levels of ADAM22 could be maintained through increased interaction with 14-3-3 following phosphorylation of ADAM22 by protein kinase A (Yokoi et al., 2021). Other studies have continued to show functional interactions between these ADAM proteins and Kv1 channels and LGI family proteins leading to regulation of axon physiology and synaptic plasticity (Lovero et al., 2015; Hivert et al., 2019; Kozar-Gillan et al., 2023; Miyazaki et al., 2024). ADAM11 has also been shown to play a role in *Xenopus* neural tube closure and cranial neural crest cell migration through its interaction with Wnt and BMP4 pathway proteins, and to serve as a ligand for integrin alpha4, thus supporting cell adhesion as do other ADAM proteins (Wang et al., 2018; Pandey et al., 2023). A variety of reports have implicated ADAM22 and ADAM23 in cancer as well. It appears that ADAM11, ADAM22, and ADAM23 utilize both their disintegrin and pseudopeptidase domains in a variety of protein-protein interactions.

## 3 M14 carboxypeptidases

**FIGURE 2:**
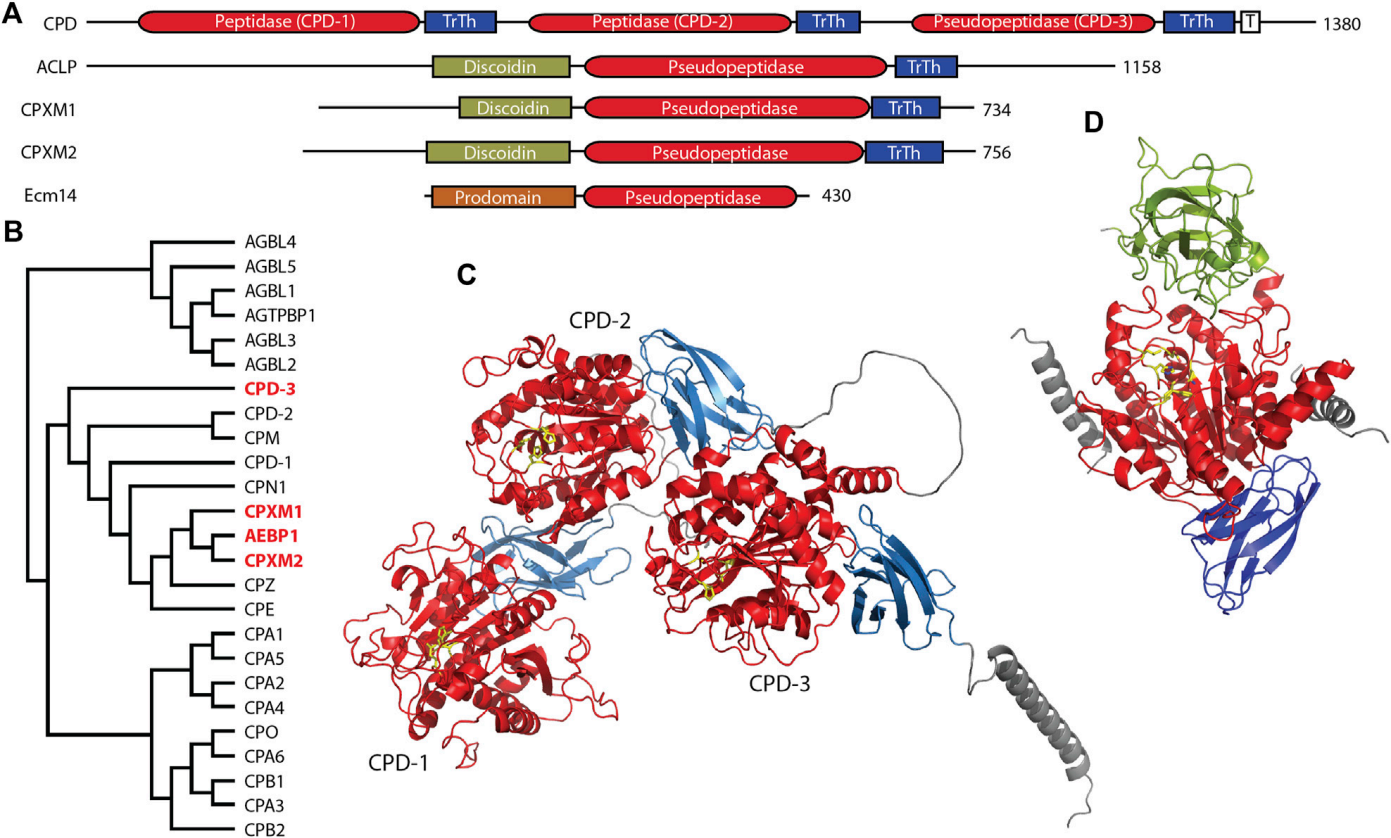
Four members of the human M14 metallocarboxypeptidase family are pseudopeptidases. **(A)** The domain structure of M14 pseudopeptidases. CPD contains 3 peptidase/transthyretin domain units and a C-terminal transmembrane segment; ACLP, CPXM1, CPXM2 all have an N-terminal discoidin domain; Ecm14 from *S. cerevisiae* is closely related to the digestive A/B carboxypeptidases with an N-terminal prodomain. **(B)** Protein sequences for human M14 metallopeptidase domains were aligned with Clustal Omega and a midpoint-rooted cladogram prepared with Iqtree2 and Dendroscope. These fall into three major groups, the cytosolic carboxypeptidases (top), the regulatory carboxypeptidases (middle) and the digestive carboxypeptidases (bottom). Pseudopeptidase members are shown in red. **(C)** The predicted structure of human CPD, as modeled by Alphafold (AF-O75976-F1-model_v4). Domains are color-coded as shown in **(A)**, and pseudoactive site residues are shown as yellow sticks. **(D)** The predicted structure of human AEBP1/ACLP as modeled by Alphafold (AF-Q8IUX7-F1-model_v4) with pseudoactive site residues shown in yellow; disordered N- and C-termini are not included. AlphaFold shows two helices (gray) derived from the N-terminal repetitive and disordered region on either side of the pseudopeptidase domain. Domains are color-coded as shown in **(A)**. Note also that the arrangement of the discoidin and carboxypeptidase domains relative to each other is predicted by AlphaFold and requires experimental validation.

The M14 family of metallocarboxypeptidases is a large family of enzymes (Figure 2B) responsible for cleaving C-terminal amino acids from substrate proteins and peptides (Reznik and Fricker, 2001; Arolas et al., 2007; Gomis-Ruth, 2008; Fernandez et al., 2010). These enzymes are commonly known to be involved in dietary protein digestion and neuropeptide processing. The family contains four pseudopeptidase members within vertebrate organisms, and one characterized pseudopeptidase in the yeast *Saccharomyces cerevisiae*, the only member of the M14 family in yeast (Figure 2A).

### 3.1 CPD, an inactive domain working with two active domains to process bioactive proteins and peptides

Carboxypeptidase D (CPD) is a protein with important roles in bioactive peptide processing (Dong et al., 1999; Varlamov et al., 1999; Zhang et al., 2008). It was discovered in a search for enzymes with carboxypeptidase E (CPE)-like activity (Song and Fricker, 1995). Both CPD and CPE are involved in the maturation of neuroendocrine peptides formed by the cleavage of long precursor proproteins by proprotein convertases (e.g., furin, PC1/3, PC2, etc.) (Zhang et al., 2008; Wardman et al., 2010). Proprotein convertases cleave at dibasic sites, often with the consensus sequence R-x-K/R-R. C-terminal basic amino acids are then removed through the action of CPE to form the fully mature peptide. Even in the absence of CPE, however, C-terminal neuroendocrine peptide processing activity remains, due to the activity of CPD (Dong et al., 1999; Fricker and Leiter, 1999; Zhang et al., 2008).

CPD is a large protein consisting of an N-terminal signal peptide, three carboxypeptidase/transthyretin domain units, and a C-terminal transmembrane segment and cytosolic tail (Figures 2A, C) (Fricker et al., 1996; Fricker, 2004). While CPD contains three tandem carboxypeptidase domains, only the first two of these are active (Tan et al., 1997); the third domain has substitutions at nearly all active-site residues (Table 2). The second domain has had its structure characterized by X-ray crystallography (Gomis-Ruth et al., 1999; Aloy et al., 2001), and AlphaFold has predicted the structure of the entire protein (Figure 2C). While this AlphaFold prediction requires experimental validation, it does present a model of how the three domains may associate. CPD expression is widely distributed throughout the body (Song and Fricker, 1996; Xin et al., 1997).

The three domains of CPD appear to have complementary functions. The active first and second domains have different specificities, with the first domain preferring C-terminal arginine and a pH of 6.3–7.5, and the second domain preferring C-terminal lysine and a pH of 5.0–6.5 (Novikova et al., 1999; Sidyelyeva and Fricker, 2002; Garcia-Pardo et al., 2017). Prior to the characterization of CPD as an enzyme, it was identified as a duck glycoprotein of 180 kDa (gp180) that bound the preS envelope protein of duck hepatitis B virus, enabling viral entry (Kuroki et al., 1995). While deletion of the first and second domains eliminated enzymatic activity, only deletion of the third domain eliminated preS binding (Eng et al., 1998). It has been suggested that this third domain may play a role in the binding of proteins and/or the presentation of substrates to the other active domains (Aloy et al., 2001), and this possibility is supported by the AlphaFold prediction (Figure 2C). Whatever this binding function may be, it has been shown to be crucial for *Drosophila* survival. A variety of transgenes expressing various domains of CPD were placed in an embryonic lethal *Drosophila* mutant, *svr* ^PG33^ (Sidyelyeva et al., 2010). It was found that both of the two active domains were necessary for viability; however, lack of the third (inactive) domain dramatically reduced this viability, when considering both survival from embryo to adult and from pupa to adult. This supports the possibility that the third domain may assist in the function of the active domains, perhaps through presentation of substrates.

### 3.2 AEBP1/ACLP, a regulator of cellular differentiation and collagen fiber properties within connective tissue

Adipocyte enhancer binding protein 1 (AEBP1) and aortic carboxypeptidase-like protein (ACLP) are both encoded by the AEBP1 gene. Both ACLP and AEBP1 contain a discoidin domain and a carboxypeptidase/transthyretin domain unit; C-terminal repetitive sequences enable AEBP1 DNA binding (Figures 2A, D) (Lyons et al., 2005; Lyons et al., 2006). Although AEBP1 was initially characterized as a transcriptional repressor that required an intact and enzymatically-active carboxypeptidase domain for this function (He et al., 1995; Muise and Ro, 1999), enzymatic activity was not detected in subsequent studies (Song and Fricker, 1997; Reznik and Fricker, 2001; Lyons et al., 2006); analysis of amino acid sequence shows that all active site amino acids are substituted except for two zinc-binding amino acids (Table 2). The nuclear AEBP1 protein isoform is derived from an alternatively-spliced or truncated mRNA (Layne et al., 1998; Ro et al., 2001), while the widely-expressed and predominant protein isoform, ACLP, contains additional repetitive amino acid sequence N-terminal to the discoidin-like domain (Figure 2A) and a signal peptide that results in its secretion and function in the extracellular matrix (Layne et al., 1998). The expression of two different proteins (AEBP1 and ACLP) with different localizations, and thus different functions, from one gene (AEBP1), presents some confusion in terminology and interpretation of experimental results, and therefore requires careful examination of the experimental details in any particular study.

The intracellular variant, AEBP1, was first identified as a transcriptional repressor that bound the AE-1 enhancer element upstream of the adipose P2 gene in preadipocytes and was downregulated upon differentiation of these cells (He et al., 1995). A similar AEBP1 cDNA was identified in an osteoblastic cell line where expression was also turned off upon final differentiation (Ohno et al., 1996). Subsequent work implicated AEBP1 in obesity and inflammation through a variety of protein-protein interactions. The gamma5 subunit of a heterotrimeric G protein was found to interact with AEBP1 via a yeast two-hybrid screen, and this interaction regulated the transcriptional repressor activity of AEBP1 at the onset of adipocyte differentiation (Park et al., 1999). Another yeast two-hybrid screen identified PTEN as an interacting partner for AEBP1 (Gorbenko et al., 2004). Co-immunoprecipitation experiments supported an interaction via the carboxypeptidase domain of AEBP1; this interaction stimulated PTEN degradation and correlated with enhanced adipose apoptosis and resistance to diet-induced obesity in AEBP1 knockout mice (Ro et al., 2007). The opposite, massive obesity, was observed in female transgenic mice, with AEBP1 overexpression in adipose tissue and macrophages (Zhang et al., 2005). AEBP1 was found to interact with the mitogen-activated protein kinases ERK1 and ERK2 through its discoidin-like domain, preventing dephosphorylation of ERK1/2 and correlating with a decreased ability of 3T3-L1 cells to undergo adipogenesis (Kim et al., 2001). Finally, *in vitro* and *ex vivo* studies identified a role for AEBP1 in regulating cholesterol efflux from macrophages and promoting foam cell formation and therefore likely playing a role in atherosclerosis (Majdalawieh et al., 2006; Majdalawieh and Ro, 2009; Huang et al., 2012). As similar results were not observed for a DNA binding-deficient mutant of AEBP1, these results were suggested to be through a transcriptional mechanism (Majdalawieh et al., 2006); subsequent work suggested that the interaction of the discoidin-like domain of AEBP1 with IκBα was also responsible (Majdalawieh et al., 2007). Further *in vivo* analysis of AEBP1 knockout mice and transgenic mice with macrophage-specific overexpression of AEBP1 showed a role for AEBP1 in the development of atherosclerotic lesions in the proximal aorta (Bogachev et al., 2011). Altogether, a variety of results have suggested a role for the intracellular AEBP1 variant in the control of cellular differentiation through the regulation of intracellular signals and transcription.

While the above data suggest possible roles for a shorter AEBP1 variant in cellular differentiation, current gene expression databases show that the primary mRNA expressed by the AEBP1 gene is the longer variant producing the ACLP protein. ACLP is highly expressed in smooth muscle cells of the aorta (Layne et al., 1998; Layne et al., 2002) and other major arteries (GTex). It is broadly expressed in collagen-rich tissues, including vasculature, dermal, and skeletal tissues, primarily in the extracellular matrix (Layne et al., 2001; Ith et al., 2005). Like AEBP1, ACLP was found to be expressed in 3T3-L1 preadipocytes and downregulated upon stimulation of differentiation (Gagnon et al., 2002). Although overexpression of ACLP in 3T3-L1 cells did not impact adipogenesis, the overexpression of ACLP in 3T3-F442A cells inhibited adipogenesis and promoted transdifferentiation into smooth muscle-like cells (Abderrahim-Ferkoune et al., 2004). Treatment of mouse and human lung fibroblasts with recombinant ACLP stimulated the fibroblast to myofibroblast transition through an upregulation of smooth muscle α-actin (SMA) and collagen expression (Tumelty et al., 2014); a similar result was obtained with mouse 10T1/2 cells and in this setting ACLP was connected with adipose tissue fibrosis (Jager et al., 2018). Other studies have implicated the AEBP1 gene and the ACLP protein in fibrosis, including lung fibrosis (Schissel et al., 2009), liver fibrosis and nonalcoholic steatohepatitis (Lou et al., 2017; Teratani et al., 2018; Gerhard et al., 2019), and renal fibrosis (Liu et al., 2023).

It is likely that ACLP interacts with collagen through its discoidin-like domain (see Figures 2A, D). Although no experiment has directly shown this, the related CPXM1 interacts with collagen in this way (Kim et al., 2015), and other discoidin domains have been shown to interact directly with collagens (Ichikawa et al., 2007). Several studies have shown the importance of ACLP-collagen interactions. When 3T3-L1 preadipocytes overexpressing ACLP were grown on collagen I-coated plates, differentiation was inhibited (Gusinjac et al., 2011). ACLP-null lung fibroblasts exhibited reduced cell spreading and proliferation when cultured on collagen; these phenotypes could be reversed upon extracellular addition of the ACLP discoidin-like domain (Schissel et al., 2009). *In vitro* experiments demonstrated that ACLP had a direct mechanical effect on collagen fibers, with collagen ACLP composite fibers exhibiting increased stiffness, toughness, and tensile strength (Vishwanath et al., 2020).

Animal models for the study of AEBP1/ACLP function, together with recent discoveries of disease connections, confirm the importance of the AEBP1 gene. Disruption of the AEBP1 gene, resulting in elimination of ACLP expression, led to the perinatal death of most homozygous null pups due to gastroschisis, the disruption of the abdominal wall (Layne et al., 2001). This gastroschisis was shown though immunohistochemistry to be a neuromuscular defect (Danzer et al., 2010). Those that survived birth had indications of defective wound healing as seen in skin lesions and reduced healing following a dermal punch (Layne et al., 2001). In another knockout mouse model, in which the gene deletion was similar, but smaller (disruption of exons 7–12, rather than exons 7–16 in the previous knockout), null offspring of heterozygous crosses were about half the expected numbers, corroborating an important role in embryonic development. In this case, however, the primary defect seen in knockouts was slower growth and decreased mass of white adipose fat pads (Ro et al., 2007). This AEBP1-null mouse also exhibited premature involution of mammary glands at late pregnancy that could be rescued by adipose-specific expression of an AEBP1 transgene (Zhang et al., 2011). It is unclear why different phenotypes were observed in these two knockout models of AEBP1 function. Although an early targeted search for AEBP1 mutations in infants affected by gastroschisis did not clearly support a connection (Feldkamp et al., 2012), a large number of recent publications have shown that mutations in the AEBP1 gene result in a subtype of autosomal recessive Ehlers-Danlos syndrome (EDS) (Alazami et al., 2016; Blackburn et al., 2018; Hebebrand et al., 2019; Ritelli et al., 2019; Syx et al., 2019; Di Giosaffatte et al., 2022; Angwin et al., 2023; Sanai et al., 2023; Yamaguchi et al., 2023). EDS is a heterogenous condition that usually presents with joint hypermobility, skin elasticity and fragility, poor wound healing, and risk of vascular rupture. EDS patients have an increased risk for abdominal wall herniation (Harrison et al., 2016), reminiscent of the gastroschisis observed in mutant mice. These disease phenotypes most closely match the observed functions for the extracellular ACLP protein isoform. The AEBP1 gene has additionally been implicated in a variety of cancers (Majdalawieh et al., 2020).

In summary, much has been published regarding AEBP1 and ACLP function. However, it is not clear what the relationship is between the various structural domains. Evidence points to a role for the discoidin domain in collagen interaction, and some studies of AEBP1 have suggested a role for the carboxypeptidase domain in other interactions. It is notable that the N-terminal unstructured region of ACLP is predicted to interact via two helices on either side of the carboxypeptidase domain, suggestive of a yet-uncharacterized regulatory function (Figure 2D). This, of course, is currently a predicted model and will need further experimental validation.

### 3.3 CPXM1 and CPXM2, collagen binding and cardiac hypertrophy

While much has been reported on the functions of AEBP1/ACLP, much less is known about the closely related proteins CPXM1 and CPXM2. Both CPXM1 and CPXM2 proteins were first identified through bioinformatics and shown to be inactive following expression in Sf9 cells and incubated with a number of artificial substrates (Xin et al., 1998; Lei et al., 1999). Sequence analysis shows that CPXM1 has a carboxypeptidase domain with a pseudo-active site very similar to the active sites of its active relatives, but for the change of the substrate-binding arginine 145 to histidine, while CPXM2 has two additional substitutions (Table 2). Similar to ACLP, both CPXM1 and CPXM2 have an inactive carboxypeptidase domain preceded by a discoidin domain and a signal peptide, so are secreted and have functions in the extracellular matrix. Both the AEBP1 and CPXM1 genes are duplicated in a wide range of fish species, while CPXM2 is not. This may be due to the nearly ten-fold larger size of the CPXM2 gene in comparison to AEBP1 and CPXM1 (Fajardo et al., 2023).

Expression of CPXM1 was found to be broad in adult tissues and high in developing mouse cartilage and skeletal structures (Lei et al., 1999); current expression databases show a preponderance of expression in fibroblasts. Using the RAW264.7 osteoclastogenesis model cell line, Chang et al. (2004) showed expression of CPXM1 in pre-osteoclasts that was downregulated upon maturation to osteoclasts. Overexpression of CPXM1 in these cells inhibited the differentiation process. Interestingly, investigation of the role of CPXM1 in adipocyte differentiation suggested an opposite role to that of AEBP1. CPXM1 was identified as a downstream effector of FGF-1 mediated proliferation and differentiation of human preadipocytes (Kim et al., 2016). Upon induction of adipocyte differentiation, CPXM1 expression increased (in contrast to the decrease of AEBP1 and ACLP expression upon 3T3-L1 differentiation (He et al., 1995; Gagnon et al., 2002; Abderrahim-Ferkoune et al., 2004)). Likewise, knockdown of CPXM1 inhibited differentiation as well as collagen expression (Kim et al., 2016). An increase in CPXM1 expression in the adipose tissue of obese mice and humans suggested a key role in adipogenesis (Kim et al., 2016), while increased CPXM1 expression in a mouse model of polycystic ovary syndrome suggested a role in the ovary as well (Pervaz et al., 2022). Further *in vitro* work through a collagen pull-down assay and mutagenesis of key amino acids within the discoidin domain showed that CPXM1 directly interacted with collagen III (Kim et al., 2015). The specific function of the pseudopeptidase domain is unknown.

CPXM2 is expressed in most tissues examined, with notably high expression in the choroid plexus (proteinatlas.org). Expression was detected by *in situ* hybridization in most regions of the mouse brain, consistent with current data from the human brain dataset (proteinatlas.com) (Xin et al., 1998). CPXM2, expressed in the Sf9 insect cell system, was able to bind to an Arg-Sepharose resin (Xin et al., 1998). It was not washed off with 200 mM NaCl, but could be eluted with 10 mM arginine, suggesting a specific interaction via its pseudo active site. This contrasted with CPXM1, which did not bind specifically to Arg-Sepharose (Lei et al., 1999). Several diseases have been connected with CPXM2, with varying levels of experimental support. The headbobber mouse contained a recessive mutation that resulted in headbobbing, circling, and deafness. This headbobber mutation was a result of transgene insertion that deleted a 648-kb segment of chromosome 7 encompassing three protein-coding genes, G protein-coupled receptor 26 (*Gpr26*), carboxypeptidase X2 (*Cpxm2*) and carbohydrate (N-acetylgalactosamine 4-sulfate 6-O) sulfotransferase 15 (*Chst15*), all expressed within the postnatal mouse inner ear and so candidates for the mutant phenotype (Somma et al., 2012; Buniello et al., 2013). Interestingly, similar mutations involving CPXM2 have been reported in humans further implicating CPXM2 and/or nearby genes in inner ear development (Miller et al., 2009; Buniello et al., 2013).

The CPXM2 gene has been associated with several diseases, many through genome-wide studies and with SNPs of unknown significance. For example, an exome sequencing and genotyping study of over 400 multiple sclerosis patients found a CPXM2 intronic polymorphism significantly associated with aggressive multiple sclerosis (Gil-Varea et al., 2018). Another genome-wide association study found a number of intronic CPXM2 SNPs associated with cognitive decline in schizophrenia (Hashimoto et al., 2013), and another found weak association with Alzheimer’s disease (Chen et al., 2016). Several studies have found association between overexpression of CPXM2 and cancers, gastric (Niu et al., 2019), hepatic (Ye et al., 2020), and osteosarcoma (Zhao et al., 2019).

Most interesting is the recent discovery of a role for CPXM2 in left ventricular cardiac hypertrophy (Grabowski et al., 2022). This study, initiated with a transcriptome analysis of the left ventricle, found an increased expression of CPXM2 in a rat hypertensive model system compared to controls. CPXM2 was upregulated in both cardiofibroblasts and cardiomyocytes, where it was found along the t-tubule network. Gene knockout in mice had a protective effect against left ventricular hypertrophy and fibrosis upon deoxycorticosterone acetate (DOCA) treatment and following 8 weeks of treatment no significant difference in left ventricular internal systolic diameter and ejection fraction was observed between DOCA-treated knockouts and sham groups. While over 1,000 transcripts were differentially expressed between wild-type sham and DOCA-treated mice, no differentially expressed transcripts were identified between knockout sham and DOCA-treated mice, suggesting an important, although currently unknown, role for CPXM2 in the mechanisms of cardiac hypertrophy.

### 3.4 Ecm14, a fungal pseudopeptidase with unknown function

The commonly used yeast model system, *S. cerevisiae*, contains one member of the M14 metallocarboxypeptidase family. The *ECM14* gene (extracellular mutant 14) was initially identified in a screen for mutations that imparted sensitivity to calcofluor white, suggesting a cell wall defect (Lussier et al., 1997). The ECM14 mutant also exhibited sensitivity to hygromycin B and papulacandin, an increased ratio of mannose to glucose (Lussier et al., 1997), and sensitivity to sonication (Ruiz et al., 1999). The Ecm14 protein is homologous to the A/B subfamily of M14 metallocarboxypeptidases (see Figure 2B). It contains a prodomain, which can be cleaved by the action of trypsin or chymotrypsin, and a domain with homology to the carboxypeptidase domain, but with a substitution of the general base glutamate for a lysine (Table 2). Likely due to this substitution, no enzymatic activity has been detected, as determined by standard assays (Figure 1A) (McDonald et al., 2020). A synthetic lethal screen in the *S. cerevisiae* system was unable to identify a function (McDonald et al., 2020). However, other studies have found Ecm14 to be involved in aggregate invasive growth in *C. albicans* and possibly in invasion by the plant pathogen *Verticillium dahlia* (Chow et al., 2019; Zhang et al., 2020). There is still much to be learned about the function of this fungal pseudopeptidase; however, the fact that the prodomain can be cleaved suggests an activation mechanism for a yet-to-be-determined function.

## 4 Other pseudometallopeptidases

Most inactive members of mammalian peptidase families are found in the M12 and M14 families. There are, however, a few reported pseudopeptidases within other peptidase families.

Tma108, a member of the **M1 aminopeptidase family**, was first identified as a translation machinery-associated factor in the yeast *S. cerevisiae* (Fleischer et al., 2006). Further work showed that Tma108 interacted with the nascent protein chain as it emerged from the ribosome (Delaveau et al., 2016). Interestingly, Tma108 only interacted with a small subgroup of about 200 nascent proteins, this group being specified by an unknown motif within the first 35 amino acids of the nascent protein. Mutagenesis of a key residue in Tma108, known to be responsible for binding of substrate N-termini in related aminopeptidases, eliminated this interaction with nascent proteins, suggesting that Tma108 utilized its substrate binding pocket to bind the nascent protein. While it was not clear if Tma108 functioned as an active enzyme [it retains all active site residues except one involved in substrate binding (Table 2)], it was shown that Tma108 remained associated with its target protein until translation was completed, suggesting that it may play a non-enzymatic role (Delaveau et al., 2016).

**FIGURE 3:**
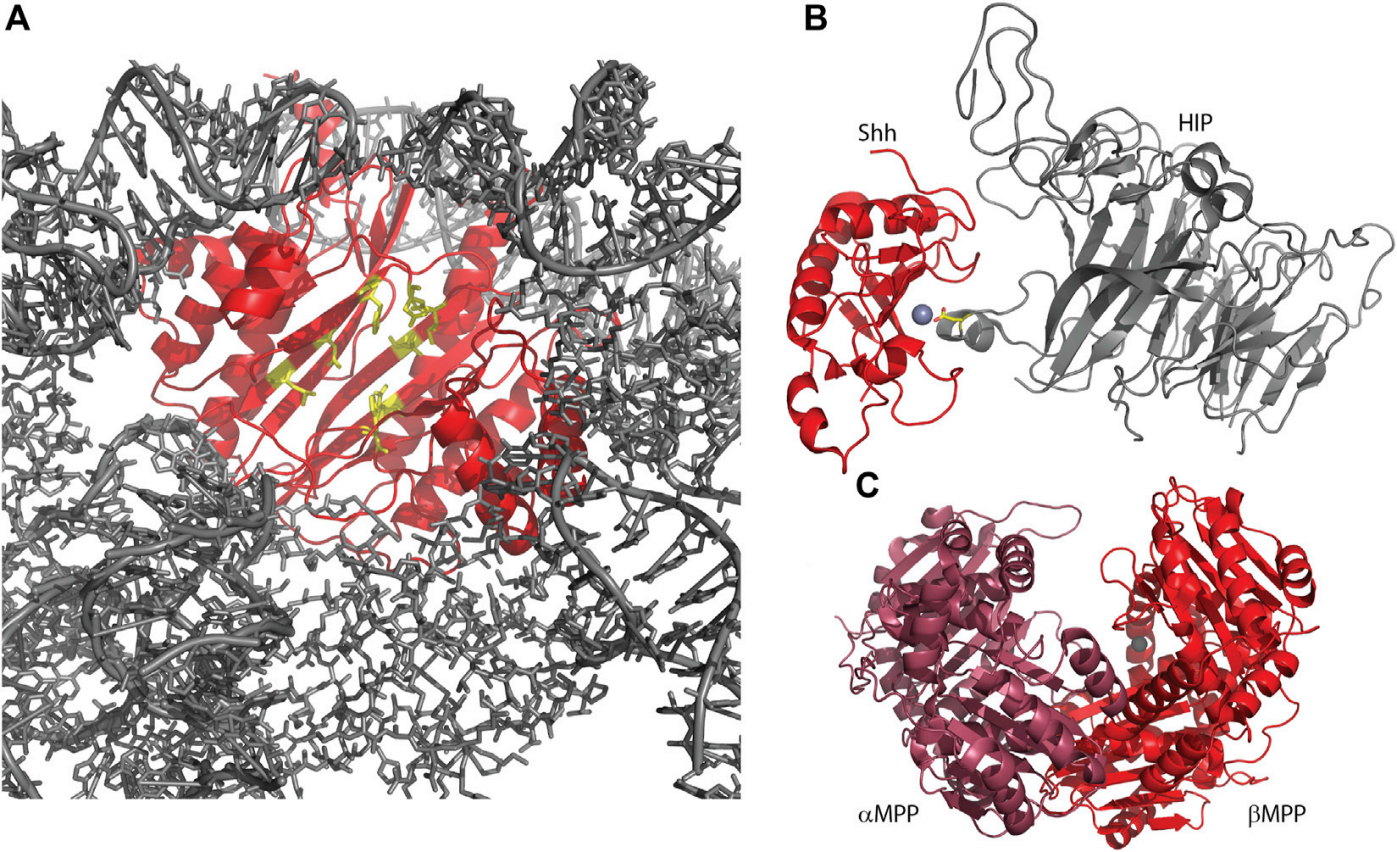
Several metallopeptidase homologs utilize their pseudo-active sites for protein interactions. **(A)** Human Ebp1, in complex with a human 80S ribosome (6SXO). The view shown is looking outwards through the ribosomal exit tunnel, surrounded by ribosomal components (gray) and capped by Ebp1 (red). Pseudo-active site residues are shown in yellow, available to capture the nascent protein N-terminus. **(B)** Sonic hedgehog protein (Shh) in complex with hedgehog-interacting protein (HIP; 2WG4) shows HIP directly interacting with the Shh-bound zinc ion via an aspartic acid (yellow sticks). **(C)** Yeast mitochondrial processing peptidase (MPP; 1HR6) is a heterodimer consisting of a beta subunit with bound zinc and an alpha subunit, lacking the necessary zinc-binding residues, but contributing other necessary residues for a functional enzyme.

ErbB3-binding protein 1 (Ebp1, or proliferation-associated 2G4, PA2G4) is another aminopeptidase homolog, within the **M24 methionine aminopeptidase (MetAP) family**, with relatively low sequence identity to the active MetAPs and retaining only two of five zinc-binding residues (Table 2) (Kowalinski et al., 2007). Ebp1 is best known for its role in cell growth and differentiation and therefore in the progression of many cancers. Depending on the context, Ebp1 can function as a tumor suppressor or as an oncogene (Stevenson et al., 2020). Ebp1 was shown to interact with a variety of RNAs, most strongly to ribosomal 5S RNA, suggesting an interaction with mature ribosomes in this manner (Kowalinski et al., 2007; Monie et al., 2007). Recently cryoEM structures of Ebp1 bound to 60S and 80S ribosomes have been reported showing Ebp1 bound to the ribosomal exit tunnel, excluding the interaction of modifying enzymes such as active MetAPs, chaperones, and targeting factors (Figure 3A) (Wild et al., 2020; Kraushar et al., 2021). The implications of this are not entirely clear, although it is likely that the presence of Ebp1 regulates the production of the nascent protein (Wild et al., 2020). The orientation of Ebp1 with the pseudoactive site toward the ribosomal exit site (Figure 3A) suggests that the MetAP active site has been repurposed for a role in guiding nascent proteins out of the ribosome, possibly playing a similar role as Tma108 (see above); however, high resolution details of the interaction between a nascent protein and Ebp1 are yet to be reported.

Gone early (Goe) is an inactive pseudopeptidase member of the **M13 neprilysin family** in *Drosophila* (Matsuoka et al., 2014), with substitutions in all key active site positions (Table 2). Goe is a type II transmembrane protein that was detected on germ cell membranes that interfaced with other germ cells, but not on neighboring somatic cells. Overexpression of Goe on germ cells resulted in a decrease in the number of primordial germ cells entering the gametogenic pathway, while decreasing Goe expression increased primordial germ cell differentiation. It was suggested that, since Goe was expressed in EGF-producing cells and was able to suppress EGFR signaling in the wing imaginal disc, Goe may have been involved in trapping EGF ligands prior to their binding of a receptor. The extracellular portion of Goe, containing the pseudopeptidase domain, was necessary for this function, while the cytosolic portion was dispensable. Interestingly, this is reminiscent of the role of the iRhom pseudopeptidases in regulating EGF signaling in *Drosophila* (see *Introduction*) (Zettl et al., 2011).

Hedgehog (Hh) protein in *Drosophila* and its mammalian homologs Sonic hedgehog (Shh), Indian hedgehog (Ihh) and Desert hedgehog (Dhh) function as secreted ligands of the Patched1 (Ptc1) transmembrane protein. This prevents interaction of Ptc1 and Smoothened (Smo) so that Smo can translocate to cilia and activate the Gli transcription factors leading to a host of developmental responses. The structure of the N-terminal signaling domain of Shh determined by X-ray crystallography showed a fold and catalytic site homologous to that of the **M15 metallopeptidases** (Hall et al., 1995; Bosanac et al., 2009) (Figure 3B; Table 2). However, mutation of putative catalytic residues H135 and E177 did not affect the cellular signaling mediated by Shh as measured via cultured cells, nor its biophysical characteristics, leading to the conclusion that Shh was a pseudopeptidase (Day et al., 1999; Fuse et al., 1999). This was further supported by crystal structures showing that hedgehog interacting protein (HIP) bound to Shh directly through the pseudoactive site of Shh (Figure 3B), while binding studies showed that peptides derived from Ptc1 and HIP, and inhibitory antibodies, bound to the pseudoactive site as well (Bishop et al., 2009; Bosanac et al., 2009; Maun et al., 2010). Recent studies, however, have called this conclusion into question, suggesting that Shh, but not *Drosophila* Hh, may in fact be an active metallopeptidase. Himmelstein et al. (2017) examined the signaling capacity of Shh and the E177A mutant *in vivo* and found that the expression of Shh-E177A at endogenous sites resulted in patterning defects similar to those seen in Shh null mice. Subsequently, Jagers and Roelink showed that zinc coordination and E177 were required for Shh association with extracellular matrix and non-cell autonomous signaling (Jägers and Roelink, 2021). They suggested that Shh might be a proteoglycan peptidase that is required for the release of Shh into the ECM; other activities of Shh *in vitro* or in cell culture systems may not require this proteolytic function.

The mitochondrial processing peptidase, MPP, is a member of the **M16B peptidase family**. MPP is a dimeric protein, composed of a proteolytically active ∼500 residue beta subunit with a zinc-binding HXXEH motif, and a homologous, yet inactive, ∼500 residue alpha subunit that lacks the zinc binding motif (Figure 3C) (Pollock et al., 1988; Taylor et al., 2001). This inactive alpha subunit is essential for MPP function, as it assists in forming the clamshell active center and contains a glycine-rich loop important for substrate binding (Table 2). In related M16A and M16C enzymes, these two subunits are combined in one ∼1,000 residue polypeptide; hence, these enzymes also have a pseudopeptidase domain that is critically important for the enzymatic function of the whole (Li et al., 2006). Prokaryotic homologs of MPP have also been identified, produced from a two-gene operon reflecting the alpha/beta subunit arrangement seen in eukaryotic MPP (Aleshin et al., 2009; Maruyama et al., 2011; Ishida et al., 2022). It is notable that the Core I and II proteins of the bc1 mitochondrial respiratory complex are homologous to alpha and beta MPP; in some cases these respiratory proteins are pseudopeptidases (Braun and Schmitz, 1995; Taylor et al., 2001).

In addition to mitochondrial function, pseudometalloproteases have also found roles within chloroplasts. The FtsHi proteins are inactive FtsH enzymes of the **M41 AAA metallopeptidase family**, with very few active site residues conserved in these members (Table 2). Active FtsH enzymes are found in both inner mitochondrial membranes and chloroplast thylakoid membranes where they assemble into hexameric complexes and use their AAA + domains to hydrolyze ATP for the translocation of substrates into a proteolytic processing chamber. In contrast, all five of the FtsHi proteins (FtsHi1-5) were found to interact with the chloroplast inner membrane translocon complex, FtsHi3 within a 1 megadalton complex, and the others within a 2 megadalton complex, where they facilitated ATP-driven protein translocation (Kikuchi et al., 2018). Although their precise roles are not known, FtsHi proteins appear to be most important in seedling development, with FtsHi1 and FtsHi4 null mutants being embryonic lethal (Mishra and Funk, 2021).

## 5 Conclusion

As might be expected, pseudopeptidases within the metallopeptidase family have taken on a diversity of biological roles *in lieu* of enzymatic activity. In some cases, the pseudopeptidases make use of the pseudoactive site for interaction rather than catalytic purposes (Tma108, Shh, possibly Ebp1 and Cpxm2). In other cases, the pseudopeptidase domain is used for interactions via other surface regions (ADAM22, ADAM23, possibly gone early). Some proteins containing pseudopeptidase domains appear to primarily use other domains in interactions, leaving the pseudopeptidase domain seemingly superfluous (ADAMs involved in sperm maturation, using their disintegrin domains for interactions; AEBP1, CPXM1, CPXM2, likely interacting with collagen via their discoidin domains). αMPP is inactive on its own but is directly involved in the enzymatic activity of its partner, βMPP. In other cases, the jury is still out on the function of the pseudopeptidase domain. In at least one case, sonic hedgehog, there remains a possibility that it is not actually a pseudopeptidase. It is likely that future work will reveal more secrets of these superficially “dead” enzymes.
